# Breastfeeding, Community Vulnerability, Resilience, and Disasters: A Snapshot of the United States Gulf Coast

**DOI:** 10.3390/ijerph191911847

**Published:** 2022-09-20

**Authors:** Tony H. Grubesic, Kelly M. Durbin

**Affiliations:** 1 Center for Geospatial Sciences, School of Public Policy, University of California at Riverside, Riverside, CA 92521, USA; 2 Childbirth International, Queenstown 9300, New Zealand

**Keywords:** breastfeeding, infant feeding, resilience, vulnerability, disasters, spatial analysis, inequity

## Abstract

Climate change-induced disasters are increasing in intensity and frequency in the United States. Infant feeding in the aftermath of an extreme event is particularly challenging, especially given large variations in community vulnerability and resilience. The aim of this study was to identify the physical, social, and spatial vulnerabilities of communities along the Gulf Coast and highlight locations where high (or low) breastfeeding initiation rates have the potential to offset (or exacerbate) infant feeding challenges in the wake of a disaster. We structured this study as a retrospective, spatial data analysis of breastfeeding initiation, the risk for extreme events, social vulnerability, and community resilience to uncover locations that may need post-disaster intervention. The results suggested that significant gaps in the geographic distribution of community risk, vulnerability, resilience, and breastfeeding initiation existed. While many metropolitan areas benefitted from high breastfeeding initiation rates, they were also the most “at risk” for disasters. Conversely, many rural communities faced less risk for extreme events but exhibited more social vulnerability and less resilience should a disaster strike. Prioritizing emergency response resources to support infant feeding after a disaster is critically important, but urban and rural communities have divergent profiles that will require variable strategies to ensure recovery. Our results highlight this variability and provide prescriptive guidance regarding where to potentially allocate emergency resources.

## 1. Introduction

Climate change-induced disasters are increasing in intensity and frequency in the United States and many other countries worldwide [1]. The effects of extreme events such as hurricanes, prolonged droughts, and other hazards negatively affect human lives. These impacts include famine, malnutrition, gastrointestinal and respiratory infections, increased mortality rates, and many forms of material deprivation [2]. Within this context, unequal societies often suffer from a vulnerability–disaster trap. In short, damages fueled by disasters can widen income inequalities and reduce resilience for groups already experiencing disenfranchisement and other forms of social vulnerability [1]. Infants are particularly vulnerable during natural hazard events because they depend on their caregivers for feeding and nutrition [3,4]. For example, if breastfeeding practices are interrupted for the mother–infant dyad during an extreme event, there is a stronger likelihood of breastfeeding cessation [5]. In addition, for infants reliant on breastmilk substitutes, there are often challenges in obtaining clean water and formula, even in highly developed locales [4]. For example, during the winter of 2021, many residents in Austin, Texas, were without their municipal water service for nearly 2 weeks after a prolonged bout of freezing weather [6]. These challenges are partly fueling the Delivering Essentials to Mothers Amid Natural Disasters (DEMAND) Act [7]. Specifically, this bill directs the Federal Emergency Management Agency (FEMA) to designate breast pumps and certain lactation supplies as eligible for financial assistance under major disaster relief provisions. It is also essential to note that recent problems with the supply chain in the US, combined with several product recalls, are making some breastmilk substitutes difficult to find—even without the impact of a major disaster [8,9].

In the United States, communities of color often face disproportionate vulnerability to disasters and hazards. For example, recent work suggested that majority Black, Hispanic, or Native American census tracts experience a 50% greater vulnerability to wildfire than other tracts in the US [10]. Similarly, undocumented Latino/a and Indigenous people are particularly vulnerable to disasters and disproportionately affected by racial discrimination, economic hardships, a lack of language proficiency, and fear of deportation before, during, and after extreme events [11]. In addition, regardless of the disaster scenario, there are often displacements of large populations, substantial resource limitations, cascading infrastructure failures, and difficulty distributing emergency resources [12]. For example, estimates suggested that 56,000 pregnant women and 75,000 infants were directly affected by Hurricane Katrina [13]. These effects included a higher percentage of preterm, low-birth-weight, and very-low-birth-weight infants in the parishes affected by the storm [13].

In recent work, Cappelli et al. [1] and Zadkovic et al. [14] suggested that these extreme events will increase in frequency and intensity. For example, Zadkovic et al. [14] noted that climate change and breastfeeding disruption share roots in a global economic system that undervalues both the environment and reproductive labor. More importantly, the lost (potential) economic value of breastfeeding “represents a missed opportunity for women to build climate resiliency, as every household dollar spent on formula or avoidable medical expenses is one not spent on other foods, healthcare, improvements to household structures, or emergency savings” [14].

This missed opportunity is particularly salient for women and children in the aftermath of disasters. For instance, the work of DeYoung et al. [15,16] and Gribble [3] confirmed several guiding principles for infant feeding during emergencies. Relatedly, Mudiyanselage et al. [2] noted four key facilitators for breastfeeding during disasters: (1) privacy (e.g., baby tents, screening curtains, shawls, and friendly spaces), (2) support of community and family networks (e.g., encouragement, financial help, assistance with childcare, and support through interpersonal communication networks), (3) adoption of professional breastfeeding support (e.g., seeking advice from health staff, NGOs, herbal healers, and traditional birth attendants), and (4) pre-existing breastfeeding practice. Where the latter is concerned, when/where breastfeeding is the cultural norm, women will continue breastfeeding during extreme events. However, Mudiyanselage et al. [2] and DeYoung et al. [15,16] also noted several challenges to breastfeeding during extreme events, including (1) decreasing self-efficacy (e.g., crisis in confidence to continue breastfeeding and belief that stress causes milk to go “bad”), (2) lack of knowledge or resources (e.g., misinformation from health professionals concerning breastfeeding, lack of food or water, and lack of privacy), and (3) over-reliance on formula (e.g., increasing availability, predatory marketing, and encouragement to use formula).

These challenges are particularly acute in disaster relief camps [17], where families need to shelter after an extreme event. Where this latter point is concerned, there are two factors worth highlighting. First, the unregulated formula distribution to disaster zones is highly problematic [3,18]. In addition to the negative relationship between breastfeeding and formula, there are often severe constraints on the availability of clean water for making the formula and generally unhygienic conditions (e.g., no means to clean bottles or safely store excess formula) [3]. Second, in communities where breastfeeding is not the cultural norm, disruptions to supply chains mean that the availability of formula, bottles, and the other equipment one needs for formula-fed infants may be nonexistent, substantially increasing the odds of infant malnutrition and adverse health outcomes. Dall’Oglio et al. [19] echoed the challenges and opportunities outlined above. However, any details relating to maternal and infant health outcomes were largely missing from the literature. In short, despite the growing interest in evidence-based interventions in humanitarian crises, most studies failed to provide sufficient empirical evidence regarding the effectiveness of these tactics and related interventions.

In this paper, we aimed to determine the effects of social vulnerability and community resilience on breastfeeding initiation in three Gulf Coast states (Louisiana, Mississippi, and Alabama)—all of which are disproportionately affected by natural hazard events, particularly hurricanes. This task is vital for highlighting communities with a potential predisposition for adverse maternal and infant outcomes should a disaster strike. However, we do not frame this as prescriptive guidance. Instead, the novelty of this paper is its use of spatially explicit approaches to explore contingencies in a “what if” framework that will give decision-makers more flexibility to adapt solutions that best fit local culture and needs.

## 2. Materials and Methods

We structure this study as a retrospective, cross-sectional exploratory and confirmatory spatial data analysis of breastfeeding initiation, risk for extreme events, social vulnerability, and community resilience. This approach helps to eliminate one-size-fits-all recommendations often found in public health studies and to uncover essential nuances between communities that might drive bespoke intervention strategies for infant feeding and mother support strategies should a disaster strike. In addition, the secondary data sources in this research did not require ethical approval.

### 2.1. Setting and Relevant Context

The Gulf Coast states of Alabama, Louisiana, and Mississippi provide an interesting setting for this research. Poverty levels in Alabama (14.9%), Mississippi (18.7%), and Louisiana (17.8%) are much greater than the US average (11.6%) [20]. The proportions of Black residents in Alabama (26.8%), Mississippi (37.9%), and Louisiana (33.1%) are also higher than the US average (14.2%) [21]. Recent data from the Centers for Disease Control and Prevention (CDC) and empirical studies suggested that all three states are laggards in breastfeeding initiation and continuation [22,23]. For example, the latest Breastfeeding Report Card from the CDC [24] (using data from infants born in 2017) includes state-level information on the percentage of breastfed infants. The national average is 84.1%. Louisiana (66.2%), Alabama (69%), and Mississippi (70%) ranked worst, second-worst, and fourth-worst, respectively, in the United States. Furthermore, in a spatial statistical analysis of breastfeeding rates in the US, Garnier et al. [22] and Grubesic and Durbin [23] identified all three states as “laggards”—far behind other locations in breastfeeding initiation, continuation, and the presence of community support resources (e.g., La Leche League and International Board Certified Lactation Consultants).

Beyond the state-level deficits in breastfeeding activity and support, all three states share a location in one of the world’s most active tropical storm basins. Since record-keeping began in 1842 [25], 187 storms have struck Mississippi, 230 have struck Louisiana, and 208 have struck Alabama. Florida is the most frequently struck state, with 443 storms making landfall since 1842. In addition to each state’s exposure to tropical storms, all three locales suffer from deeply entrenched poverty, and they harbor numerous, high-risk, vector-borne tropical diseases (e.g., Dengue Fever), a propensity for inadequate housing (often without plumbing), and underdeveloped transportation infrastructure, particularly in rural areas [26,27].

### 2.2. Sample

**Figure 1:**
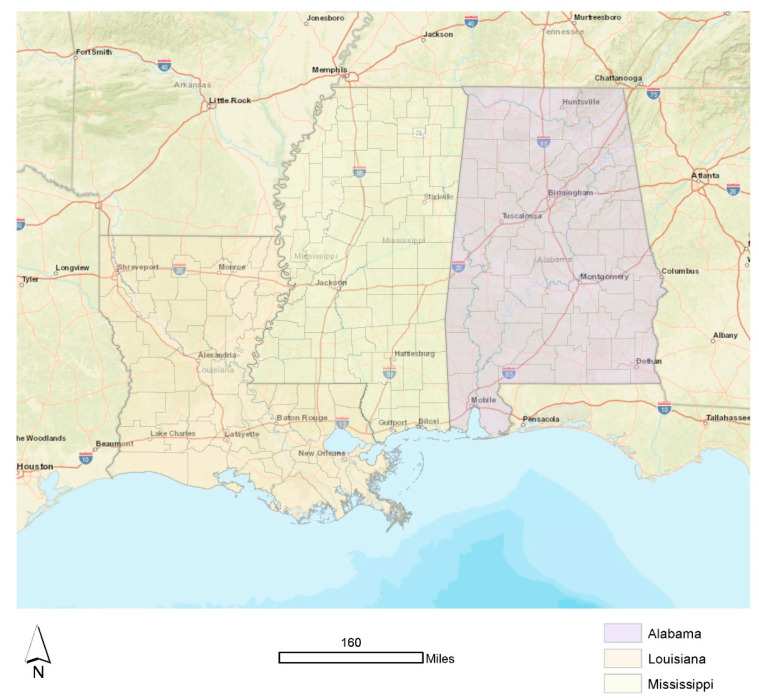
Study area: Louisiana, Mississippi, and Alabama.

The target population for this study was residents of the states of Alabama, Louisiana, and Mississippi. To clarify, we were not interested in tracking individuals, their breastfeeding outcomes, or disaggregate measures of household-level risk, vulnerability, or resilience. However, we were interested in evaluating these factors at the community level. In sum, there were 213 counties in our study area: 67 in Alabama, 82 in Mississippi, and 64 (parishes, a county equivalent) in Louisiana (Figure 1). The study area included a total of 140,776 square miles with a total population of 12,280,405 residents.

### 2.3. Measurement and Data Analysis

We obtained a geographic database of all Alabama, Louisiana, and Mississippi counties from the Environmental Systems Research Institute [28]. In addition, we collected breastfeeding initiation rates for each county from the CDC [29]. These rates draw upon US birth certificate breastfeeding initiation data from 2018 to 2019. Specifically, the CDC defines breastfeeding initiation as “any infant that received milk or colostrum between delivery and discharge from a birth facility or completion of birth certificate for home births” [29]. We also obtained a current list of Baby-Friendly hospitals for the region from Baby-Friendly USA [30]. We converted this into a dummy variable for statistical modeling where county *i* = 1 if a Baby-Friendly hospital is present and 0 otherwise.

Next, we obtained county-level risk data from the Federal Emergency Management Administration (FEMA) National Risk Index (NRI) [31]. Supporting technical documentation is also available from FEMA [32]. The NRI covers natural hazards ranging from coastal flooding and hail to volcanic activity and tropical storms. The NRI considers a total of 18 different hazard types for analysis. Ultimately, each county receives a score ranging from 0 to 100. The final risk score for each county represents a community’s risk relative to all other counties in the United States. Under the hood, the score is representative of a composite value for all 18 hazard types, a social vulnerability index (SOVI) [33], a community resilience index (BRIC) [34], and a measure for expected annual losses (EAL) in a community. The final risk score for each county undergoes a min–max normalization process, enhancing interpretability and its distributional characteristics [32]. For clarity, we represent risk in each county using the following general equation:

*Risk* = *Expected Annual Loss* × *Social Vulnerability* × 1/*(Community Resilience).* (1)

As detailed above, we drew the constituent components for the risk index from various sources. Firstly, we used the social vulnerability index [33], which comprises 29 socioeconomic variables that reflect a community’s ability to prepare for, respond to, and recover from hazards. These data include measures for vulnerable populations (e.g., elderly, children under 5 years of age, and women), income (e.g., poverty and affluence), demography (e.g., Black, Hispanic, etc.), healthcare infrastructure (e.g., hospitals per capita), and housing status [32,33]. The SOVI ranges from 0 (very low) to 100 (very high).

Secondly, we used the community resilience index (BRIC) [35], which comprises 49 variables in six categories, including (1) human wellbeing/cultural/social (e.g., educational attainment, English competency, health insurance, and food security), (2) economic/financial (e.g., employment and income inequality), (3) infrastructure/built environment/housing (e.g., housing quality, industrial resupply potential, and evacuation routes), (4) institutional/governance (e.g., flood insurance coverage, jurisdictional fragmentation, and disaster training), (5) community capacity (e.g., social networks, volunteerism, and political engagement), and (6) environmental/natural (e.g., local food supplies, water stress, and energy use) categories. Given the six categories, BRIC’s range is limited, with values from 41.1 (very low) to 64.7 (very high) [33,35].

Lastly, the expected annual loss (EAL) index represents the average annual economic loss (in USD) resulting from natural hazards. Specifically, EAL accounts for losses using several consequence types (e.g., buildings, population, and agriculture). For example, extreme drought has no direct effects on buildings; hence, the EAL index captures the drought effects on crops and agriculture (i.e., losses). EAL uses a Value of Statistical Life (VSL) approach for population impacts. VSL recognizes 7.6 million USD of economic loss for each fatality or 10 injuries. FEMA converts all historical losses to 2020 USD (for more details on calculating expected annual loss, see [32]).

This study used cross-sectional confirmatory spatial statistical methods and exploratory spatial data analysis. The exploratory elements of this work consisted of geovisualization techniques to identify geographic patterns for each variable of interest, including breastfeeding initiation, social vulnerability, community resilience, and risk. The exploratory analysis also included the application of the classic k-means clustering approach [36,37] to identify peer communities for both risk and breastfeeding initiation in the study area.

We used confirmatory statistical analysis in this paper to identify associations among breastfeeding initiation, social vulnerability, community resilience, risk (via estimated annual losses), and Baby-Friendly hospitals. Specifically, we used a spatial error model with a queen’s spatial weights matrix to capture the local context of breastfeeding initiation, risk, resilience, and vulnerability. We opted for spatial models over the classic ordinary least squares regression (OLS) to better account for the underlying spatial dependence in the county-level cross-sectional data [23,38]. In this instance, the spatial error model accounts for the dependency of residual errors for an observation of interest (e.g., county) with companion errors in the local neighborhood (i.e., contiguous counties). The choice of a spatial error model also reflects if it is likely that we are missing some unknown but potentially important variables for explaining breastfeeding initiation rates.

## 3. Results

### 3.1. Exploratory Data Analysis

**Figure 2:**
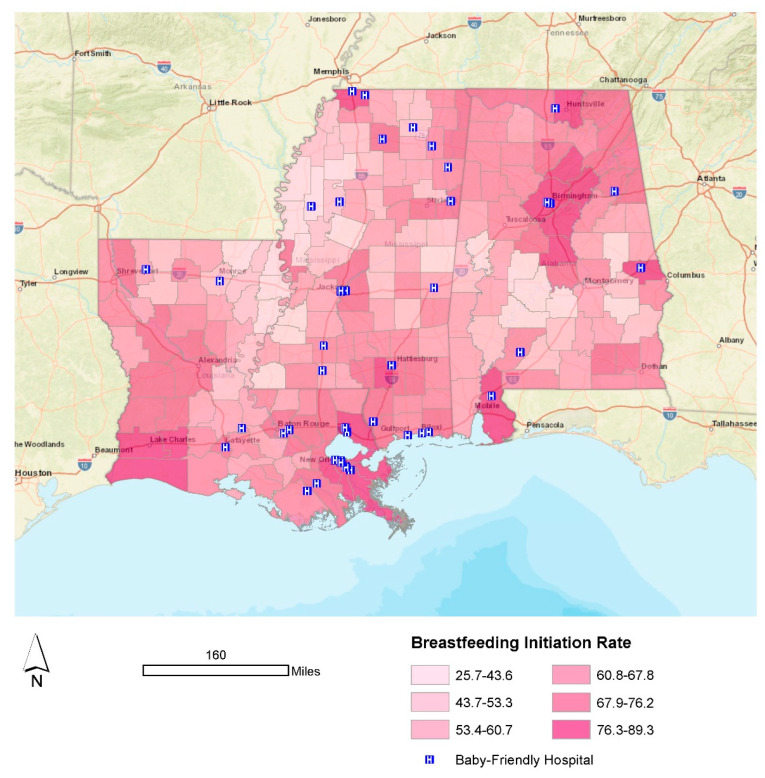
Breastfeeding initiation rate: 2018–2019.

We display the study area and its breastfeeding initiation rates by county/parish in Figure 2. Urban areas exhibited the highest levels of breastfeeding initiation, with Shelby County, Alabama (89.3%) leading the way and East Carroll Parish, Louisiana (25.7%) trailing all other locations. We also use Figure 2 to highlight the geographic distribution of all Baby-Friendly hospitals in the region. Most of these hospitals can be found in larger metropolitan areas (although not exclusively), including New Orleans, Birmingham, Baton Rouge, and suburban Memphis (Desoto County, MS, USA).

**Figure 3:**
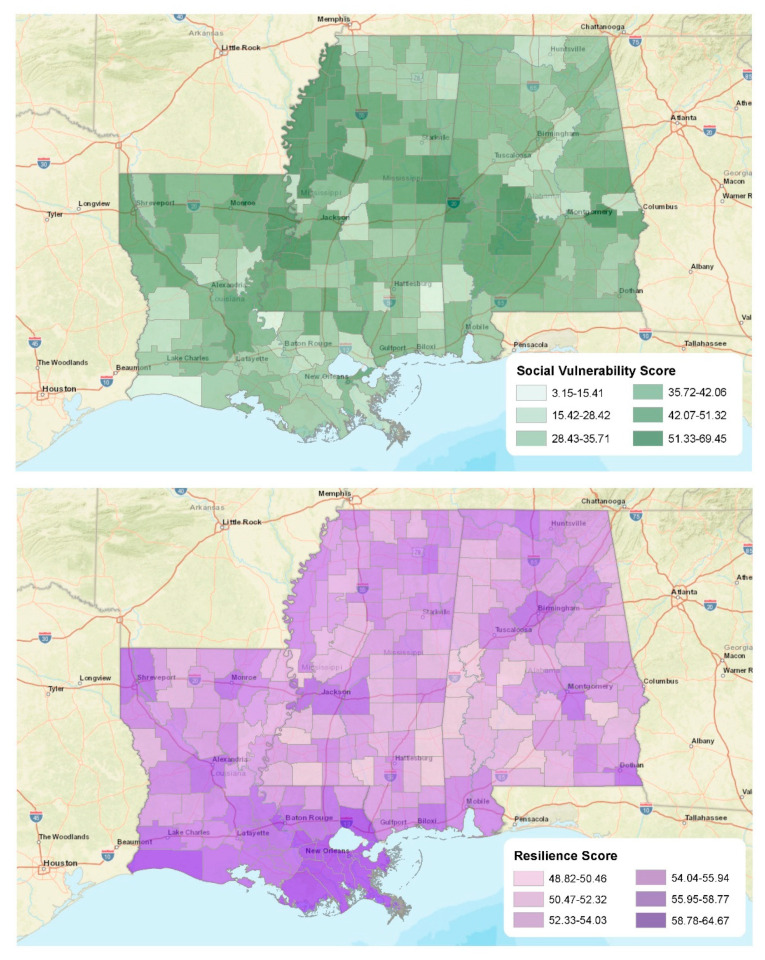
Social vulnerability and resilience scores.

**Figure 4:**
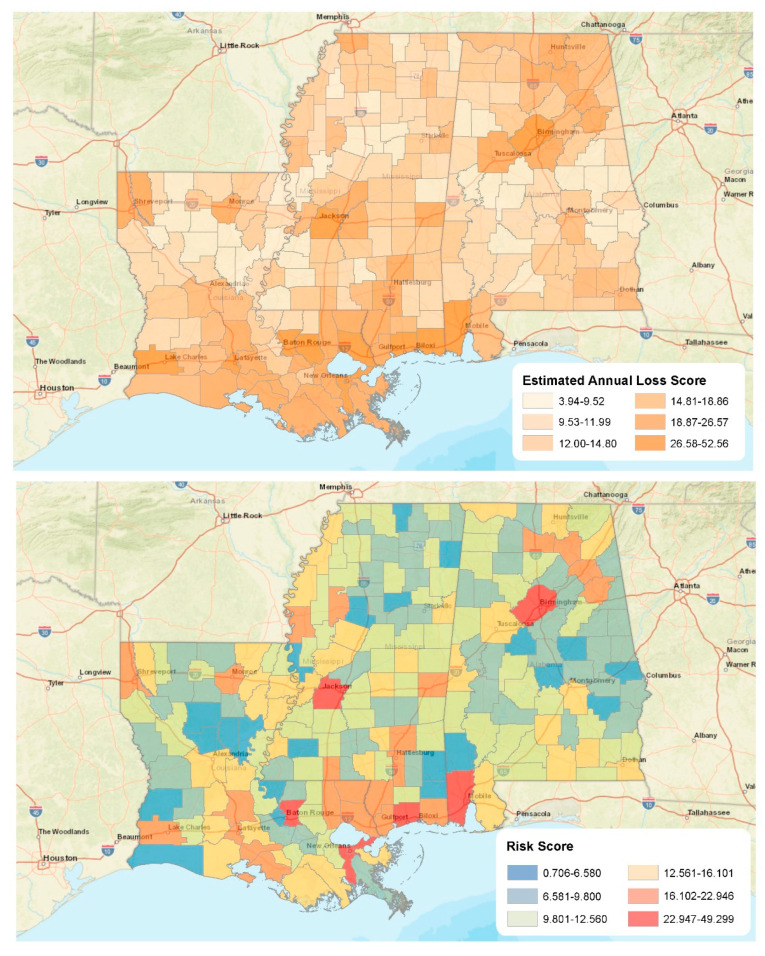
Expected annual loss and composite risk scores.

We provide the descriptive statistics for key study indicators in Table 1, but illustrate their distributions in Figure 3 and Figure 4. For instance, we highlight each county’s social vulnerability and community resilience scores in Figure 3. There are several patterns worth noting here. First, levels of social vulnerability were exceptionally high along the Mississippi River (which forms the western border of Mississippi and portions of the eastern border for Louisiana). For example, these highly vulnerable communities include Bolivar, Coahoma, Tunica, and Washington counties in Mississippi and Tensas and Madison parishes in Louisiana. However, the geographic distribution of resilience for the study area tells a different story. In this instance, one can find the most resilient communities in more urbanized counties and parishes, including those in/around New Orleans, Shreveport, Jackson, Montgomery, and Birmingham. Again, this result does not suggest that these counties are impervious to adverse outcomes after an extreme event. However, it does suggest that more social, economic, and community capital exists in these urban locations, along with higher-quality housing and infrastructure systems.

In many ways, Figure 4 offers a complimentary snapshot of urban dominance. The estimated annual loss scores were highest in counties in our study area’s major metropolitan regions. This pattern makes sense when considering the densities of residential housing, infrastructure, commercial, and industrial properties in these locations. When disasters strike these locales, they damage more high-value assets compared to rural areas. Again, using Katrina as a contextual touchstone, the storm caused more than 160 billion USD in damage in 2005 [39]. The composite risk score for our study area confirms this. Reconsidering Equation (1) from above, when one combines risk (EAL) with social vulnerability (SOVI) and community resilience (BRIC), there are a handful of urban locations where the risk composite is quite high, including Orleans (49.29) and East Baton Rouge (26.93) parishes, Mobile (32.65) and Jefferson (28.06) counties in Alabama, and Harrison County, Mississippi (30.74).

**Figure 5:**
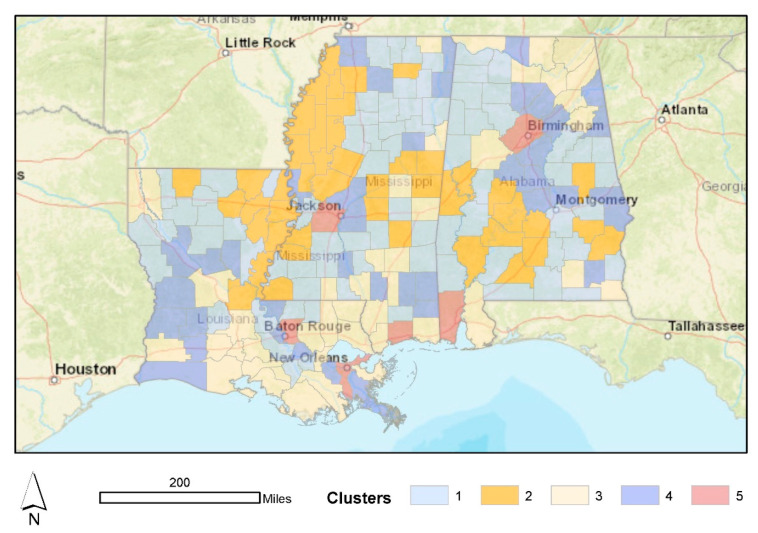
Clusters of breastfeeding initiation and risk.

We connect breastfeeding initiation rates with the composite risk indicator via k-means clustering in Figure 5 and Table 2. Specifically, we display the cluster analysis results in Table 2, along with average risk scores and breastfeeding initiation rates for each county/parish in the study area in Figure 4. We used a cluster scheme with five groups for analysis because it offered the best balance for the ratio of *between* to *total sum of squares*. While all of the clusters are interesting in their own right, two groups are especially worth highlighting. First, Cluster 5 represents urban counties and parishes (*n* = 7) that suffered from the highest average risk scores but exhibited relatively high breastfeeding initiation rates relative to the region (Table 2). Conversely, Cluster 2 represents those (*n* = 45) that exhibited a modest risk for extreme events but also displayed the lowest average breastfeeding initiation rates in the study region (Table 2). Cluster 2 represents a mix of primarily rural and micropolitan areas throughout all three states but mainly concentrated along the Mississippi River in Louisiana and Mississippi and southwest of Montgomery, Alabama. Recall that these are many areas that suffer from high levels of social vulnerability (Figure 2).

### 3.2. Confirmatory Data Analysis

We detail the cross-sectional spatial error model results for our study region in Table 3. Model 1 was our base model and included three independent variables: (1) social vulnerability, (2) community resiliency, and (3) Baby-Friendly hospitals (dummy). Model 2 added the estimated annual loss variable. Again, we used a queen-based contiguity measure for the spatial error model, but sensitivity analysis with alternative spatial weights matrices (e.g., distance-based) yielded no substantial changes to the model results, variable coefficients, or interpretation. Model 1 suggests that social vulnerability has a statistically significant but negative effect on breastfeeding initiation. For every one-unit increase in the SOVI, we see, on average, a decrease of 0.60% in breastfeeding initiation.

Conversely, community resilience has a statistically significant and positive effect on breastfeeding. For every one-unit increase in BRIC, we see, on average, a 1.15% increase in breastfeeding initiation. The hospital metric was also statistically significant and suggested that counties and parishes with a Baby-Friendly hospital, on average, improve breastfeeding initiation by 3.6%. Lastly, Lambda was significant and positive, suggesting that spatial autocorrelation is present in model errors. With the addition of estimated annual losses (EAL), Model 2 represented an overall improvement in fit (AIC = 1436 vs. 1444). In short, using EAL better isolated the effects of large urban areas on the model. In this instance, the coefficient for social vulnerability remained negative. Community resilience remained positive, although its contribution to breastfeeding initiation was more muted. The Baby-Friendly hospital metric was no longer significant, but this is because the majority of them appeared in urban areas where EAL values were the highest. In short, they explained variance in similar ways. That said, for every one-unit increase in EAL, we see, on average, a 0.31% increase in breastfeeding initiation.

## 4. Discussion

Several facets of these results are worth further discussion. Firstly, there are many communities in Louisiana (e.g., East Carroll and Madison parishes), Mississippi (e.g., Bolivar and Claiborne counties), and Alabama (e.g., Wilcox and Bullock counties), among others, that struggle with breastfeeding initiation. This finding is not novel. However, given the socioeconomic and demographic composition of the region, especially within many counties exhibiting lower breastfeeding initiation rates, the known barriers for Black women and breastfeeding are likely playing a role [40,41], but these barriers are not insurmountable [42,43]. The Black population for all six locations detailed above is greater than 60% [21]. Asiodu et al. [42] suggested that paying more attention to the challenges, needs, and wants of community members is critical, as well as efforts to empower BIPOC organizations for empowering change [42]. Secondly, the results of this work strongly suggest that, while urban areas in this region suffer from elevated levels of disaster risk, the underlying community resources that support breastfeeding initiation are substantial. For example, reconsider Figure 1 and the results for Model 1. The presence of a Baby-Friendly hospital has a substantial positive effect on breastfeeding initiation, but most of these hospitals appear in urban areas. From a public health perspective, these results suggest that improving the geographic distribution of Baby-Friendly hospitals to more rural and remote areas can substantially improve breastfeeding rates in this region. One imperfect example is South Sunflower County Hospital in Mississippi. With a Black population approaching 70%, Sunflower County has a breastfeeding initiation rate of 40.1%. While this may seem low compared to more urban counties in Mississippi (e.g., Lamar County has an initiation rate of 80.1%), when comparing Sunflower County to several of its neighboring, majority-Black counties, including Bolivar (34.8%) and Washington (37.5%), it is clear that the Baby-Friendly hospital is making a positive impact within this largely rural community. It is also important to acknowledge that previous work suggested that many community-based support resources for breastfeeding favor urban areas [44], including La Leche League and Breastfeeding USA peer support networks. Thus, future analysis should include a more detailed exploration of these alternative support structures for the region, especially its rural areas.

A third facet of the results highlights the many communities with moderate risk exposure and low breastfeeding initiation rates (Table 3; Figure 5). These Cluster 2 counties and parishes are primarily rural, but there are micropolitan areas within these locales. These communities are the best candidates for investments in breastfeeding education and allied community support efforts. It is also important to remember that most of these counties exhibit very low levels of community resilience. Thus, the most vulnerable populations in these communities, including mothers and infants, are likely to suffer disproportionately if a large disaster strikes. Again, communities lacking a breastfeeding culture and local expertise for breastfeeding support will likely depend on artificial formula for feeding infants during a disaster, which is a poor outcome for the mother–infant dyad [17,19]. Furthermore, the confirmatory statistical analysis suggested that investments in community resilience, including food security, employment, housing quality, flood insurance, political engagement, and healthcare, can make a big difference in breastfeeding initiation. Again, these communities are ripe for efforts to improve resilience, reduce vulnerabilities, and educate parents on the benefits of breastfeeding.

### Limitations

There were several limitations to this study worth detailing. Firstly, the results of this analysis are representative at the county level only. Pockets of breastfeeding initiation variability likely exist within each county. Furthermore, we are certain that social vulnerability, community resilience, and risk vary within each county. Higher-resolution data, perhaps at the Census tract level, could provide additional insights into these local geographic variations. Secondly, it is essential to reaffirm that vulnerability, resilience, and risk are moving targets. Climate change, state political regimes, healthcare market dynamics, and many other factors can affect how communities prepare, plan for, absorb, recover from, and adapt to disasters [31]. This dynamism is especially salient for vulnerable populations, including mothers and infants. As a result, readers should consider these results as a current snapshot of the region. Things will change.

## 5. Conclusions

Our results suggested a strong connection between breastfeeding initiation rates and social vulnerability, community resilience, and risk in Louisiana, Mississippi, and Alabama. Social vulnerability has a statistically significant and negative effect on breastfeeding initiation, while community resilience has a statistically significant and positive effect on breastfeeding initiation. When conceptualized as expected annual losses, risk also positively affected breastfeeding initiation, but this is an artefact of urbanity. In short, the most at-risk communities are large urban areas, but these are also the locations where a depth of breastfeeding support resources exist, and this is reflected in the higher levels of breastfeeding initiation in urban communities throughout the study area. Furthermore, our results (Model 1) suggested that the presence of at least one Baby-Friendly hospital has a strong, positive effect on breastfeeding initiation. Lastly, it is essential to acknowledge substantial opportunities throughout the study region to improve breastfeeding initiation and potentially continuation outcomes. The cluster analysis revealed many communities with low levels of breastfeeding initiation yet moderate levels of risk for disasters. These locations are potentially ripe for education programs, additional Baby-Friendly hospitals, or community-based efforts to enhance local breastfeeding support structures. These efforts might include International Board Certified Lactation Consultants (IBCLCs), La Leche League, Breastfeeding USA, Baby Café, or telelactation programs if quality (and affordable) internet services are available [45,46].

## Figures and Tables

**Table 1:** Descriptive statistics for key indicators.

	*N*	Min	Mean	Max	SD
Breastfeeding initiation	213	25.70	60.96	89.30	11.87
Social vulnerability score	213	3.15	38.67	69.45	9.88
Community resilience score	213	48.82	53.93	64.67	2.713.93
Estimated annual loss score	213	3.93	14.74	52.55	7.16
Overall risk score	213	0.70	11.76	49.29	5.41
Baby-Friendly hospitals (dummy)	44	-----	-----	-----	-----

**Table 2:** Cluster attributes for breastfeeding initiation and composite risk.

Cluster	Average Risk Score	Average Breastfeeding Initiation Rate	Within-Cluster Sum of Squares
Cluster 1 (Low Risk, Moderate Initiation) [*n* = 77]	9.689	58.98	21.674
Cluster 2 (Moderate Risk, Low Initiation) [*n* = 45]	11.945	44.213	26.65
Cluster 3 (Moderate Risk, Moderate Initiation) [*n* = 43]	15.93	68.379	25.585
Cluster 4 (Low Risk, High Initiation) [*n* = 43]	7.703	73.49	19.936
Cluster 5 (High Risk, High Initiation) [*n* = 7]	31.605	71.471	17.902
Within-Cluster Sum of Squares	111.749		
Between Cluster Sum of Squares	312.251		
Ratio of Between to Total Sum of Squares	0.736		
Total Sum of Squares	424		

**Table 3:** Spatial error models results for the relationship between breastfeeding initiation and community risk, vulnerability, and resilience indicators.

Independent Variable	Model 1 (Vulnerability, Resilience, and Support)	Model 2 (Vulnerability, Resilience, Risk, and Support
Constant	21.5889 (0.103)	39.002 (0.005) *
Social vulnerability	−0.6085 (0.000) *	−0.6248 (0.000) *
Community resilience	1.1568 (0.000) *	0.7642 (0.004) *
Baby-Friendly hospitals (dummy)	3.6437 (0.005) *	1.6677 (0.246)
Estimated annual loss	-----	0.3184 (0.001) *
Lambda	0.5950 (0.000) *	0.5578 (0.000) *
R-squared	0.6763	0.6869
AIC	1443.61	1436.08
Degrees of freedom	209	208
Breusch–Pagan test	7.7159 (0.052)	14.0037 (0.021)

* Significant at the 0.05 level.

## Data Availability

All data are publicly available.
